# IGF-I and IGFBP-3 and the risk of lung cancer: A meta-analysis based on nested case-control studies

**DOI:** 10.1186/1756-9966-28-89

**Published:** 2009-06-24

**Authors:** Bo Chen, Shan Liu, Wei Xu, Xueli Wang, Weihong Zhao, Jianqing Wu

**Affiliations:** 1 Department of Geriatrics, The First Affiliated Hospital, Nanjing Medical University, Nanjing, PR China

## Abstract

**Background:**

Lung cancer is the leading cause of death from cancer worldwide. Conventional studies mainly think that insulin-like growth factor-I (IGF-I) and IGF-binding protein-3 (IGFBP-3) may promote and inhibit tumor growth, respectively. However, there are many different results about their function in some recent epidemiological studies. To evaluate the relationship between circulating serum levels of IGF-I, IGFBP-3 and lung cancer, a systematic review and meta-analysis of the published data was performed.

**Methods:**

Literatures searched on PubMed and Embase databases were enrolled in the Meta-analysis. The Meta-analysis of all eligible studies was applied with Stata 10.0 software, and the pooled odds ratio(OR) and weighted mean difference (WMD) value were obtained. The Q test, Egger's test and Begg's funnel plot were used to evaluate the heterogeneity and publication bias between the studies.

**Results:**

There are no statistically significant heterogeneity and publication bias between the studies. For IGF- I, the pooled OR and WMD were 0.87(95%CI: 0.60~1.13,) and -3.04(95%CI: -7.10~1.02, P = 0.14), respectively. For IGFBP-3, the pooled OR and WMD were 0.68(95%CI: 0.48~0.88,) and -112.28(95%CI: -165.88~-58.68, P < 0.0001), respectively.

**Conclusion:**

The association between circulating IGF- I levels and the risk of lung cancer were not statistically significant; IGFBP-3, acts as a tumor suppressor and has a inverse correlation with the risk of lung cancer.

## Background

Lung cancer is the leading cause of death from cancer worldwide, and the care rate remains less than 15% despite improvements in surgery, radiotherapy and chemotherapy [1]. Insulin-like growth factor-I (IGF-I) and IGF-binding protein-3 (IGFBP-3) have been widely accepted that they may have key role on the genesis and development of many types of tumor including lung cancer [2-7].

Growth hormone stimulates production of IGF-I in the liver and peripheral tissues. IGF-I is also released locally in response to damage, either directly or through the action of other factors associated with tissue responses to damage, including epidermal growth factor, fibroblast growth factor, and platelet-derived growth factor [8]. IGFBP-3 is the dominant circulating binding partner for both IGFs, accounting for 70 to 80% of their blood levels [8,9]. Multiple lines of evidence suggest involvement of the IGF pathway across a range of malignancies, including both non-small cell lung cancer (NSCLC) and small cell lung cancer [5,10,11]. Elevated plasma levels of IGF-I have been associated with an increased risk of lung cancer, and high plasma levels of IGFBP-3 associated with a reduced risk [5]. Similarly, IGFBP-3 promoter methylation in tumor cells has been linked to decreased survival in stage I NSCLC patients. These suggest that IGF-I may promote tumor cell growth, while IGFBP-3 acts as a tumor suppressor gene [12,13]. At the same time, different results were obtained from other studies. Recently, many large-scale clinical prospective case-control studies on association between circulating levels of IGF-I, IGFBP-3 and the risk of lung cancer were performed [14-19]. However, the results of these studies still remain inconclusive, partially because of the possible relatively small sample size in each of the published studies.

Here we performed a systematic meta-analysis of all studies published to date to determine and assess the strength of the association between circulating levels of IGF- I and IGFBP-3 and lung cancer. It may be helpful in the diagnosis and treatment of lung cancer.

## Methods

### Search strategy and study selection

PubMed and Embase were searched using the search terms: "insulin-like growth factor-I", "lung neoplasm", "case-control study", "cohort study" and "prospective study" (last search was updated on 1 March 2009). All eligible studies were retrieved, and their bibliographies were checked for other relevant publications. Review articles and bibliographies of other relevant studies identified were hand-searched to find additional eligible studies. These searches were restricted to studies in which IGF-I and IGFBP-3 concentration were measured. Two investigators independently reviewed all potentially relevant articles. Disagreement or uncertainty between 2 investigators was resolved by discussion. Inclusion was restricted to **nested case-control **studies and **prospective cohort **studies published in English.

### Data extraction

Data were independently abstracted in duplicate by 2 investigators using a standard protocol and data-collection form. Characteristics abstracted from the studies included name of the first author, location of the study, year of publication, case definition, control definition, selection criteria, method of IGF-I and IGFBP-3 measurement, confounding factors that were controlled for by matching or adjustment and mean and standard deviation (SD) of IGF-I and IGFBP-3 in each group, odds ratio (OR) comparing the highest category to the lowest and its 95% confidence interval(CI). For data not provided in tabular form or the main text, the required information were obtained by contacting corresponding authors as possible as we can.

### Statistical analysis

Most of studies provided crude and adjusted OR. We used the adjusted OR comparing the highest category with the lowest as the principal effect measure in our meta-analysis. The cutoff values for these categories were based on control groups, which better represented the distribution of IGF-I and IGFBP-3 in the general population. The adjusted ORs and their 95% confidence intervals were abstracted directly from the publications. We also used the weighted mean difference (WMD) to compare circulating levels of IGF-1 and IGFBP-3 of lung cancer cases with that of their controls.

Heterogeneity assumption was checked by the chi-square-based Q test [20]. A P value > 0.10 for the Q test indicates a lack of heterogeneity among studies, so the pooled OR estimate of the each study was calculated by the fixed-effects model (the Mantel-Haenszel method) [21]. Otherwise, the random- effects model (the DerSimonian and Laird method) was used [22]. An estimate of potential publication bias was carried out by the funnel plot, in which the standard error of log (OR) of each study was plotted against its log (OR). An asymmetric plot suggests a possible publication bias. Funnel plot asymmetry was assessed by the method of Egger's linear regression test, a linear regression approach to measure funnel plot asymmetry on the natural logarithm scale of the OR. The significance of the intercept was determined by the t test suggested by Egger (P < 0.05 was considered representative of statistically significant publication bias) [23]. Stata statistical package version 10.0(Stata Corporation, College Station, TX) was used for the meta-analysis, using two-sided P-values.

## Results

### Characteristics of studies included in the meta-analysis

Twenty-two articles were identified by the PubMed and Embase databases search. After reading abstracts and full text of them, 6 articles met the inclusion criteria. All of them are nested case-control within cohort studies as shown in Table 1. Among the 6 studies, 2 studies were conducted in the United States and 4 were done in China, Japan, Finland and British. The number of cases and controls ranged from 93 to 230 and 186 to 9,351, respectively. The total numbers of cases and controls in these studies were 1,043 and 11,472.

**Table 1:** Characteristics of case-control studies for lung cancer and IGF-I and IGFBP-3

Study	Year, location	Sample size (case/control)	Measurement		OR(95%CI) for IGF-I	OR(95%CI) for IGFBP-3	Adjusted factors in the model in original report
						
			IGF-1	IGFBP3			
Lukanova et al.[14]	2001, USA	93/186	RIA	RIA	0.54(0.14–2.07)	0.90(0.28–2.85)	Age, date of recruitment in the study, menopausal status, current smoking, time since last meal, cotinine and BMI
London et al.[15]	2002, China	230/740	RIA	IRMA	0.86(0.47–1.57)	0.50(0.25–1.02)	Smoking
Spitz et al.[16]	2002, USA	159/297	ELISA	ELISA	0.64(0.31–1.33)	2.35(1.13–4.92)	Age, sex, race, year of enrollment, and year of blood draw, BMI, smoking status, pack-years of smoking, exposure population
Waikai et al.[17]	2002, Japan	194/9351	IRMA	IRMA	1.74(1.08–2.81)	0.67(0.45–1.01)	Age, area, gender, smoking habits, and BMI
Ahn et al.[18]	2006, Finland	200/400	ELISA	ELISA	0.76(0.39–1.49)	0.71(0.35–1.47)	Age, intervention arm, BMI, and years of smoking
Morris et al. [19]	2006, British	167/498	ELISA	ELISA	1.21(0.62–2.35)	1.70(0.87–3.30)	Age, smoking

All are nested case-control studies within cohort study.

BMI indicates body mass index.

IRMA, immunoradiometric assay; ELISA, enzyme-linked immunoabsorbent assay; RIA, radioimmunoassay assay.

### Statistical heterogeneity

After performing the tests for heterogeneity for IGF-I and IGFBP-3 separately, we decided to use a fixed-effect model to obtain a summary statistic as the tests were not statistically significant (Q-value of 5.86 with df = 5, P = 0.320 for IGF-I and Q-value of 6.66 with df = 5, P = 0.247 for IGFBP-3).

### Meta-analysis results

We abstracted OR comparing the highest category to the lowest and its 95% CI and mean, SD of IGF-I and IGFBP-3, separately. And the data are shown in Table 1 and Table 2.

**Table 2:** Studies reporting IGF-I and IGFBP-3 levels in lung cancer patients and their controls

Serum factors	References	Cases			Cases		
			
		N1	Mean(ng/ml)	SD(ng/ml)	N2	Mean(ng/ml)	SD(ng/ml)
IGF-1	[14]	93	---	---	186	---	---
	[15]	230	123	46.43	740	127	41.62
	[16]	159	158	56	297	153	54
	[17]	194	124	54	9351	126	57
	[18]	200	137.2	52.3	400	145.5	52
	[19]	167	---	---	498	---	---

IGFBP-3	[14]	93	---	---	186	---	---
	[15]	230	1793	487.43	740	1863	458.76
	[16]	159	30700	8200	297	29400	7900
	[17]	194	2780	860	9351	2990	810
	[18]	200	2228	650	400	2369	640
	[19]	167	---	---	498	---	---

N1 is the number of cases, N2 is the number of controls; ---, not available.

**Figure 1:**
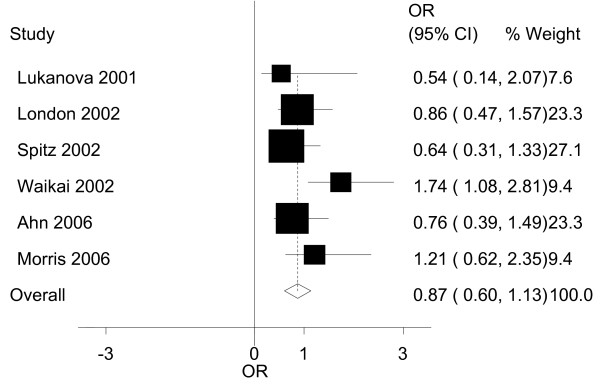
**Graphic representation of the meta-analysis for IGF-I and lung cancer**. The ORs and their 95% confidence intervals in the original studies are shown..

**Figure 2:**
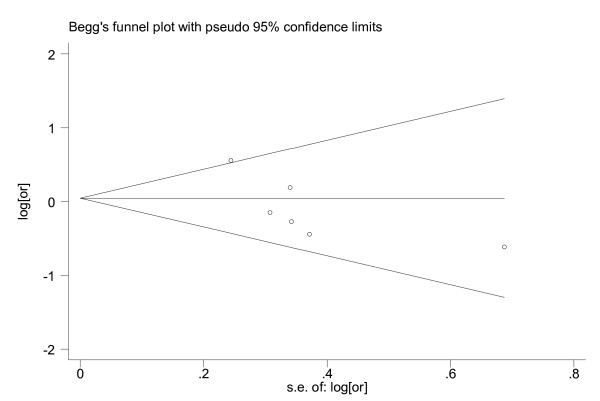
**Funnel plot for publication bias in the analysis of IGF-I and lung cancer**. Each circle indicates the logarithm of the odds ratio of lung cancer comparing the subjects in the highest category with the lowest (vertical axis) and the standard error of logarithm of odds ratio in each study. The line in the centre indicates the summary diagnostic odds ratio.

For IGF-I, the results of our meta-analysis and its graphic plot are presented in Table 3 and Figure 1. While comparing the highest to the lowest levels of IGF-I in all the studies, the people in the highest strata had a 0.87(95%CI: 0.60~1.13) times higher risk of developing lung cancer. This association was not found to be statistically significant. Both the Egger's test and Begg's funnel plot did not show any publication bias (P = 0.102; Figure 2).

**Table 3:** Individual and combined WMD, ORs and 95% CIs by IGF-I and IGFBP-3

References	IGF-1		IGFBP-3	
		
	WMD(95%CI)	OR(95%CI)	WMD(95%CI)	OR(95%CI)
[14]	---	0.54(0.14,2.07)	---	0.90(0.28,2.85)
[15]	-4.00(-10.71,2.71)	0.86(0.47,1.57)	-70.00(-141.14,1.14)	0.50(0.25,1.02)
[16]	5.00(-5.65,15.65)	0.64(0.3,11.33)	1300.00(-259.41,2859.41)	2.35(1.13,4.92)
[17]	-2.00(-9.69,5.69)	1.74(1.08,2.81)	-210.00(-332.13,-87.87)	0.67(0.45,1.01)
[18]	-8.30(-17.16,0.56)	0.76(0.39,1.49)	-141.00(-250.77,-31.23)	0.71(0.35,1.47)
[19]	---	1.21(0.62,2.35)	---	1.70(0.87,3.30)

Totol effect	-3.04(-7.10,1.02)	0.87(0.60,1.13)	-112.28(-165.88,-58.68)	0.68(0.48,0.88)

---, not available.

**Figure 3:**
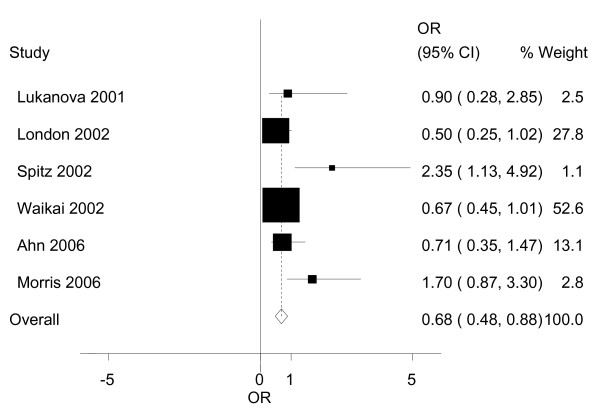
**Graphic representation of the meta-analysis for IGFBP-3 and lung cancer**. The ORs and their 95% confidence intervals in the original studies are shown..

**Figure 4:**
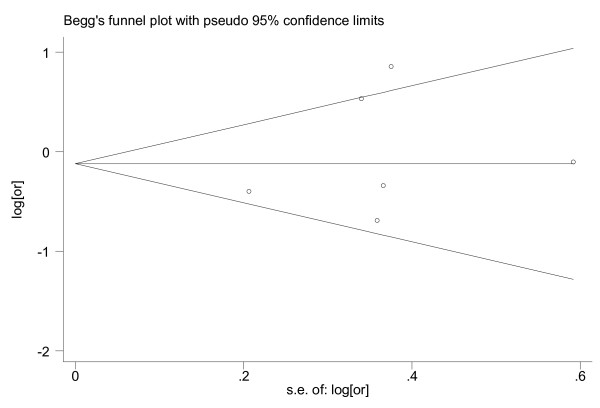
**Funnel plot for publication bias in the analysis of IGFBP-3 and lung cancer**. Each circle indicates the logarithm of the odds ratio of lung cancer comparing the subjects in the highest category with the lowest (vertical axis) and the standard error of logarithm of odds ratio in each study. The line in the centre indicates the summary diagnostic odds ratio.

We also examined the possible association of IGFBP-3 and the risk of lung cancer as presented in Table 3 and Figure 3. When we compared the highest to the lowest levels of IGFBP-3, the people in the highest strata had a 0.68(95%CI: 0.48~0.88) times higher risk of developing breast cancer. The association was statistically significant. Similarly, we also did not find any publication bias between the studies (P = 0.502; Figure 4).

The individual and combined WMD of IGF-I and IGFBP-3 are shown in Table 3. We compared circulating levels of IGF-I and IGFBP-3 of lung cancer cases with that of controls, the results are the overall WMD = -3.04(95%CI: -7.10~1.02, P = 0.14) for IGF-I, and WMD = -112.28(95%CI: -165.88~-58.68, P < 0.0001) for IGFBP-3. The publication bias were also not statisitically significant and the funnel plot were not shown.

### Sensitive analysis

A single study involved in the meta-analysis was deleted each time to reflect the influence of the individual data-set to the pooled ORs, and the corresponding pooled ORs were not materially altered (data not shown).

## Discussion

Lung cancer is the leading cause of malignancy-related mortality. The mechanism of carcinogenesis is very complex, which involves many factors, such as IGF-I and IGFBP-3. Conventional studies coordinately think that IGF-I and IGFBP-3 may promote and inhibit tumor growth, respectively. In recent years, there are many epidemiological studies have different results. In this meta-analysis, our data suggests that IGF-I low in the lung cancer population, though we could not demonstrate statistical significance. With regard to the association between IGFBP-3 and lung caner, the data suggests IGFBP-3 acts as a tumor suppressor and has a inverse correlation with the risk of lung cancer, and it does have statistical significance.

The IGF family is supposed to play a pivotal role in regulating cell proliferation, apoptosis and transformation [24]. Most circulating IGFs are produced by hepatocytes in response to growth hormone stimulation [25-27]. Circulating IGFBP-3 is produced by hepatic endothelium and Kupffer cells [26,27]. A number of in vitro and in vivo studies have demonstrated that IGF-I is an effective mitogen in normal epithelial cells and has strong antiapoptotic effects on lung cancer cells [5,10,11]. However, the effect of IGF-I may be modulated by IGFBP-3 in circulation because most of the IGF-I is bound to IGFBP-3 and once bound it is not in its active form. The results of this meta-analiysis indicate that there are no statistically significant association between IGF-I and lung cancer, while the associaton between IGFBP-3 and lung cancer is very significant. High serum levels of IGFBP-3 associated with a reduced lung cancer risk.

Lung cancer is a multifactorial disease that results from complex interactions between many genetic and environmental factors. This means that there will not be single gene or single environmental factor that has large effects on lung cancer susceptibility. Due to limited studies and published data in original report, we could not make subgroup analysis by sex, smoking status, histological types and other variables. Possible limitations of our meta-analysis includes relatively small number of studies, different heterogeneous matching factors, different countries and ethnicities, possible publication bias, as well as possible interaction with other biologic and environmental factors. It is well documented that ethnic factor contributes to the lung cancer incidence. In our study, we included 2 U.S., 1 Chinese, 1 Japanese, 1 Finnish and 1 British studies. Therefore, heterogeneity by ethnicity needs to be taken into account when interpreting our data. Heterogeneous matching factors and differential adjustment for confounding factors are other sources of bias. The above limitations might have contributed to the low statistical power of our meta-analysis.

Despite some limitations, our results based on nested case-control studies which represent of best study design. In addition, we obtained the results from dichotomous and continuous variable respectively, which made the results more reliable. What's more, heterogeneity and publication bias of the studies were not significant. Thus, the data of our study are reliability.

## Conclusion

In summary, we found that association between circulating levels of IGF-I, IGFBP-3 and the risk of lung cancer are marginally and statistically significant, respectively. So it may be helpful in the diagnosis and treatment of lung cancer. Since circulating IGF-I and IGFBP-3 remain important factors in lung cancer, more studies need to be conducted to discern this association. And uniform adjustment of confounding factors across the studies will help in terms of interpretability and comparability.

## Competing interests

The authors declare that they have no competing interests.

## Authors' contributions

In our study, all authors are in agreement with the content of the manuscript. Each author's contribution to the paper: BC: First author, background literature search, data analysis, development of final manuscript. JQW: Corresponding author, research instruction, data analysis, development of final manuscript. SL: background literature search, data analysis. WX: data analysis, background literature search. XLW: research instruction, background literature search. WHZ: research instruction, development of final manuscript.
